# Melatonin ameliorates myocardial injury by reducing apoptosis and autophagy of cardiomyocytes in a rat cardiopulmonary bypass model

**DOI:** 10.7717/peerj.11264

**Published:** 2021-04-15

**Authors:** Xiaolin Huang, Jian Hou, Suiqing Huang, Kangni Feng, Yuan Yue, Huayang Li, Shaojie Huang, Mengya Liang, Guangxian Chen, Zhongkai Wu

**Affiliations:** 1 Department of Cardiac Surgery, First Affiliated Hospital of Sun Yat-Sen University, Guangzhou, China; 2 NHC Key Laboratory of Assisted Circulation, Sun Yat-sen University, Guangzhou, China

**Keywords:** Cardiopulmonary bypass, Melatonin, Myocardial protection, Apoptosis, Autophagy

## Abstract

**Background:**

Myocardial injury is a frequent complication after cardiac surgery with cardiopulmonary bypass (CPB). This study aimed to test the hypothesis that melatonin could attenuate myocardial injury in a rat CPB model.

**Methods:**

Eighteen male Sprague-Dawley rats were randomly divided into three groups, *n* = 6 for each group: the sham operation (SO) group, CPB group and melatonin group. Rats in the SO group underwent cannulation without CPB, rats in CPB group intraperitoneal injected an equal volume of vehicle daily for 7 days before being subjected to CPB and rats in melatonin group intraperitoneal injected 20 mg/kg of melatonin solution daily for 7 days before being subjected to CPB. After 120 min for CPB, the expression levels of plasma interleukin (IL) -6, IL-1β, superoxide dismutase (SOD), glutathione peroxidase (GSH-Px), malondialdehyde (MDA), creatine kinase (CK) -MB and cardiac troponin T (cTnT) were measured. Reactive oxygen species (ROS) were detected by dihydroethidium (DHE). Apoptosis was detected by terminal deoxynucleotidyl transferase dUTP nick-end labeling (TUNEL) staining. Mitochondrial damage and autophagosomes were detected by electron microscopy. Apoptosis inducing factor (AIF) was detected by immunofluorescence. The expression of B cell lymphoma/leukemia2 associated X (Bax), B cell lymphoma/leukemia 2 (Bcl-2), cytochrome C (Cyto-C), cleaved caspase-9, AKT, p-AKT, signal transducer and activator of transcription 3 (STAT3), p-STAT3, LC3, P62, mechanistic target of rapamycin kinase (mTOR), p-mTOR and glyceraldehyde-3-phosphate dehydrogenase (GAPDH) were determined using western blotting.

**Results:**

Melatonin significantly decreased the levels of IL-1β, IL-6, MDA, CK-MB and cTnT and increased the levels of SOD and GSH-Px, all of which were altered by CPB. Melatonin reduced cardiomyocyte superoxide production, the apoptosis index and autophagy in cardiomyocytes induced by CPB. The AKT, STAT3 and mTOR signaling pathways were activated by melatonin during CPB.

**Conclusion:**

Melatonin may serve as a cardioprotective factor in CPB by inhibiting oxidative damage, apoptosis and autophagy. The AKT, STAT3 and mTOR signaling pathways were involved in this process.

## Introduction

Cardiopulmonary bypass (CPB) is widely used in most cardiac surgeries and in the treatment of other diseases. However, CPB is aggressive and can result in a high degree of injury to organs, such as heart (Whitlock et al., 2015), lungs (Hirao et al., 2017), brain (Von Rhein et al., 2014), kidneys (Belley-Cote et al., 2016), and intestines (Adamik et al., 2017). Thus, an increasing number of studies have focused on organ protection after CPB, especially myocardial protection. In addition to prompt coronary revascularization to reduce myocardial ischemia time, antiapoptotic and anti-autophagic medication is considered an effective potential therapy by cardiovascular surgeons (Khoynezhad, Jalali & Tortolani, 2004).

Melatonin (N-acetyl-5-methoxytryptamine) is a highly conserved molecule, mainly produced by the pineal gland and mitochondrial-rich organs such as the heart (Semak et al., 2005; Tan et al., 2013), liver and brain (Reiter et al., 2017; Venegas et al., 2012). Because of its antioxidant properties, it is regarded as an antiapoptotic and anti-autophagy medication (Pi et al., 2015; Reiter et al., 2018; Zhang & Zhang, 2014). Previous studies demonstrated that melatonin has significant effects on ischemia-reperfusion (I/R) injury, myocardial chronic intermittent hypoxia injury, pulmonary hypertension, vascular diseases, valvular heart diseases, and lipid metabolism, suggesting its potential as a new therapeutic option for cardiovascular disease (Sun, Gusdon & Qu, 2016). Pretreatment with melatonin increased cell survival by activating a series of signaling pathways, thus leading to a reduction in mitochondrial and cellular oxidative stress, mitochondrial fission, endoplasmic reticulum stress, and apoptosis after cardiac I/R injury (Singhanat et al., 2018). However, the protective effects of melatonin against CPB-induced myocardial injury are still little know, although there is some evidence regarding its effect on decreasing the renal and hepatic damage induced by CPB (Huang et al., 2008; Wang et al., 2009).

In the present study, the effect of melatonin on preventing CPB-induced myocardial injury was investigated in a rat model, and the downstream regulatory mechanism of its function was also studied.

## Materials & Methods

### Animals

Approximately 12-week-old healthy male Sprague-Dawley (SD) rats (450–500 g) were obtained from the Laboratory Animal Center of Sun Yat-sen University (Guangzhou, China, SYXK 2015-0107). All animals were maintained in cages under constant temperature (22 ± 2 °C), humidity (45 ± 5%), a 12 h day and 12 h night cycle, and they were given standard rodent chow and water ad libitum. There was no enrichment provided throughout the study. All rats were euthanized by intraperitoneal injection of pentobarbital sodium (50 mg/kg) to avoid or limit pain/distress. The rats were also euthanized if, (1) severe body weight loss up to 10% in 1 week; (2) animal showing no inclination to feed or drink; (3) animal can’t tolerate the experiment. However, no rats were euthanized prior to the planned end of the experiment. Any surviving rats at the conclusion of the experiment were euthanized. All animal protocols were approved by the Institutional Animal Care and Use Committee, Sun Yat-Sen University, Guangzhou, China (SYSU-IACUC-2020-000058), and were performed in accordance with the NIH guidelines (Guide for the Care and Use of Laboratory Animals).

### Animal grouping and administration

A total number of 18 rats were randomly divided into three groups, *n* = 6 for each group: the sham operation (SO) group, CPB group and melatonin group. Rats in the SO group underwent cannulation without CPB, rats in CPB group intraperitoneal injected an equal volume of vehicle daily for 7 days before being subjected to CPB and rats in melatonin group intraperitoneal injected 20 mg/kg of melatonin solution daily for 7 days before being subjected to CPB. The melatonin administration protocol (dose, way and duration of administration) in this study was determined based on previous studies (Di et al., 2020; Huang et al., 2008; Wang et al., 2009). Melatonin (Sangon Biotech, Shanghai, China) was initially dissolved in ethanol and then diluted in sterile water (final concentration of ethanol <5%).

### Surgical procedure and sample collection

The rat model of CPB was generated as previously described with some modifications (Dong et al., 2005). Briefly, individual SD rats were intraperitoneally administered pentobarbital (50 mg/kg), and additional pentobarbital was used to maintain anesthesia. Respiration was maintained by lung ventilation (Harvard Apparatus, Holliston, MA, USA) via a 16-G tracheotomy tube. The right femoral vein was cannulated with a 20-gauge heparinized catheter (Becton Dickinson Medical Devices, Suzhou, China), which was followed by systemic administration of heparin (250 U/kg). The right femoral artery was cannulated with a 22-gauge heparinized catheter for arterial infusion via the CPB circuit. The left femoral artery was cannulated with a 22-gauge heparinized catheter to monitor arterial pressure and to collect arterial blood for arterial blood gas analysis. An 18-gauge catheter was inserted into the right jugular vein. The mini-CPB circuit consisted of a venous reservoir, a roller pump (Longer Precision Pump, Baoding, China), a specially designed membrane oxygenator (Kewei Medical Instrument, Dongguan, China), and sterile tubing with an inner diameter of two mm for the venous and arterial lines. The CPB circuit was primed with a total volume of 10 mL synthetic colloid solution (8.5 mL sterile hydroxyethyl starch and 1.5 mL 5% NaHCO_3_). A flow rate of 100 mL/(kg/min) was maintained during cardiopulmonary bypass. After 120 min for CPB, the remaining priming solution was transferred into the rat, the cannulas were removed, and the incisions were sutured. The sham control rats were anesthetized, ventilated, and cannulated but did not receive CPB. Post-CPB monitoring lasted for 120 min, and then all the animals were euthanized.

Blood samples were collected from the right jugular vein via a drainage tube after cannulation (T0), at the end of CPB (T1), and 120 min after operation (T2). Approximately 0.6 mL of blood was collected at each time point. The plasma was obtained by centrifugation and stored at −80 °C before detection. The left ventricle tissues were sampled after euthanized for further evaluation.

**Figure 1:**
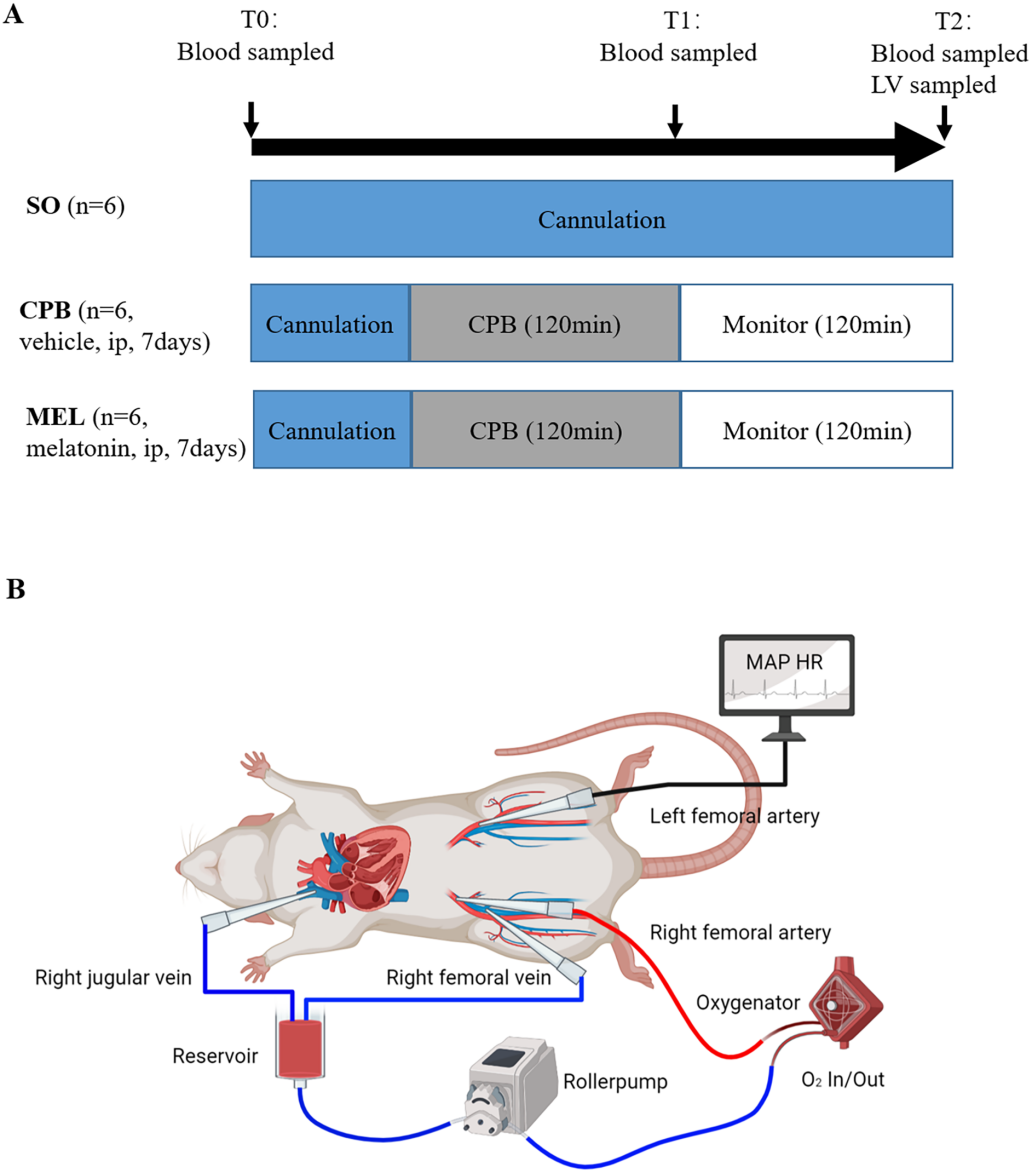
Experimental protocol (A) and schematic diagram of the CPB model (B). Experimental protocol (A) and schematic diagram of the CPB model (B). T0: after cannulation; T1: at the end of CPB; T2: 120 min after operation. SO, sham operation; CPB, cardiopulmonary bypass; MEL, melatonin; ip, intraperitoneal; MAP, mean arterial pressure; HR, heart rate; LV, left ventricle.

A detailed diagram of the experimental procedure is presented in Fig. 1.

### Echocardiography

Cardiac function was assessed in conscious rats by using transthoracic echocardiography (VisualSonics system, Toronto, Ontario, Canada), which was performed after cannulation (T0), at the end of CPB (T1), and 120 min after operation (T2). M-mode and two-dimensional echocardiography were performed to assess cardiac parameters, including ejection fraction of the left ventricle, fractional shortening of the left ventricle, wall thickness, left ventricular internal diameter, left ventricular mass, and left ventricular volume. Echocardiography data were analyzed by investigators blinded to treatment and genotype. The average of at least three measurements was used for each single data point.

### Plasma sample detection

The plasma levels of interleukin (IL)-6, IL-1β and cardiac troponin T (cTnT) were analyzed according to the instructions of the commercial rat-specific enzyme-linked immunosorbent assay kit (Elabscience Biotechnology, Wuhan, China). The plasma levels of creatine kinase (CK)-MB were measured by an automated analyzer (Chemray 800, Rayto Life and Analytical Sciences, Shenzhen, China). The plasma levels of superoxide dismutase (SOD), glutathione peroxidase (GSH-Px) and malondialdehyde (MDA) were measured using colorimetric kits (Jiancheng Institute of Bioengineering, Nanjing, China) according to the manufacturer’s instructions.

### In situ superoxide detection

Dihydroethidium (DHE) was used to assay the production of Reactive oxygen species (ROS) in situ as described previously (Chan et al., 2007). Briefly, the slides of unfixed tissue from different groups were incubated with DHE (Sigma, 10 *μ*mol/L) in PBS at room temperature for 30 min. Then, the slides were washed, fixed, mounted, and subjected to fluorescence microscopic analysis (Carl Zeiss Microscopy, LLC, Thornwood, NY, USA).

### Histologic analysis

The heart tissues were fixed in 4% paraformaldehyde, embedded in paraffin and sectioned at a thickness of 5 µm. In situ apoptosis was examined by the terminal deoxynucleotidyl transferase dUTP nick-end labeling (TUNEL) staining method using an In-Situ Cell Death Detection Kit (Fluorescein) (Roche Applied Science, Mannheim, Germany) as reported previously (Yue et al., 2017). Briefly, the slices were washed three times in 0.01 M PBS and then permeabilized in proteinase K for 10 min. After an additional three washes, the sections were incubated in TdT buffer at 37 °C for 1 h and then with antibody at 37 °C for 1 h. Afterward, 4 Elzverts, 6-diamino-2-phenylindole (DAPI) was used to stain the cell nuclei. The TUNEL/DAPI-positive cells represented apoptotic cardiomyocytes. Sections were randomly selected, and five areas were randomly selected from each section. The percentage of TUNEL-stained positive nuclei = number of TUNEL-positive nuclei/total nuclei ×100. Apoptotic nuclei were quantified by counting the total number of TUNEL-positive nuclei in an entire section from 6 different rat hearts per group.

The immunofluorescence staining procedure was reported previously (Hou et al., 2020). Immunofluorescence staining of apoptosis inducing factor (AIF) was performed using a primary antibody against AIF (Cell Signaling Technology, Danvers, MA, USA) and an Alexa Fluor 568 secondary antibody (Invitrogen, Shanghai, China). Finally, the nuclei were stained with DAPI (Sigma-Aldrich, St. Louis, MO, USA).

Images were captured by a Zeiss digital camera connected to a Zeiss VivaTome microscope (Carl Zeiss Microscopy, LLC, Thornwood, NY, USA).

### Fluorescence intensity analysis

Data were collected as previously described in Hou et al. (2020). Immunohistochemical expression was evaluated using Image-Pro Plus 6.0 software (Media Cybernetics, Silver Spring, Maryland, USA). In brief, at least five fields with positive expression in a section of myocardial tissue were randomly selected, and then these positive regions were analyzed with Image-Pro Plus 6.0 software to determine their integral optical density and area. The average of the optical density values, which represented the expression intensity in the section, was subsequently calculated.

### Electron microscopy

The technique used was described previously (Mei et al., 2018). The specimen was immersed in 2.5% glutaraldehyde and then postfixed in 2% osmium tetroxide in sodium phosphate buffer for 2 h at 4 ° C. The samples were dehydrated in a graded series of ethanol and propylene oxide. Then, all samples were embedded in araldite. One-micrometer sections were cut with an ultramicrotome (Leica EM UC7; Leica, Nussloch, Germany). After staining with lead citrate and uranyl acetate, the sections were observed using a Hitachi Transmission Electron Microscope (HT7700; Hitachi, Tokyo, Japan).

### Western blotting

The technique used was described previously (Hou et al., 2020). Proteins were isolated from heart tissues with lysis buffer (Beyotime Institute of Biotechnology, Shanghai, China) that included a protease inhibitor cocktail (Millipore, Billerica, Massachusetts, USA). Proteins were subjected to sodium dodecyl sulfate-polyacrylamide gel electrophoresis (SDS-PAGE) and transferred to polyvinylidene difluoride (PVDF) membranes (Millipore, Billerica, Massachusetts, USA). Primary antibodies against LC3, p-AKT, AKT, p-mTOR, mTOR, p-STAT3, STAT3, Bax (Cell Signaling Technology, Danvers, MA, USA), P62, Bcl-2, Cytochrome C and Caspase-9 (Abcam, Cambridge, MA, USA) were used. Subsequently, the membranes were incubated with an HRP-conjugated secondary antibody (Thermo Fisher Scientific, Waltham, MA, USA) at room temperature for 1 h, and antigen-antibody complexes were detected by a western blotting luminol reagent (Sigma-Aldrich). Glyceraldehyde-3-phosphate dehydrogenase (GAPDH) (Proteintech, Rosemont, IL, USA) served as an internal reference. ImageJ software was used to analyze the mean light density of each band. The expression of target genes was normalized to that of GAPDH.

### Statistical analysis

Data are presented as the mean ± Standard Error of Mean (SEM). Statistical analysis was performed using SPSS v. 22.0 (IBM Corp, Armonk, NY, USA). The differences in the data between the two groups were determined by Student’s t test. Comparisons among groups were performed using one-way ANOVA followed by Tukey’s post hoc test. For all tests, *p* < 0.05 was considered statistically significant.

## Results

### Pretreatment with melatonin protects the heart from CPB-induced injury

**Figure 2:**
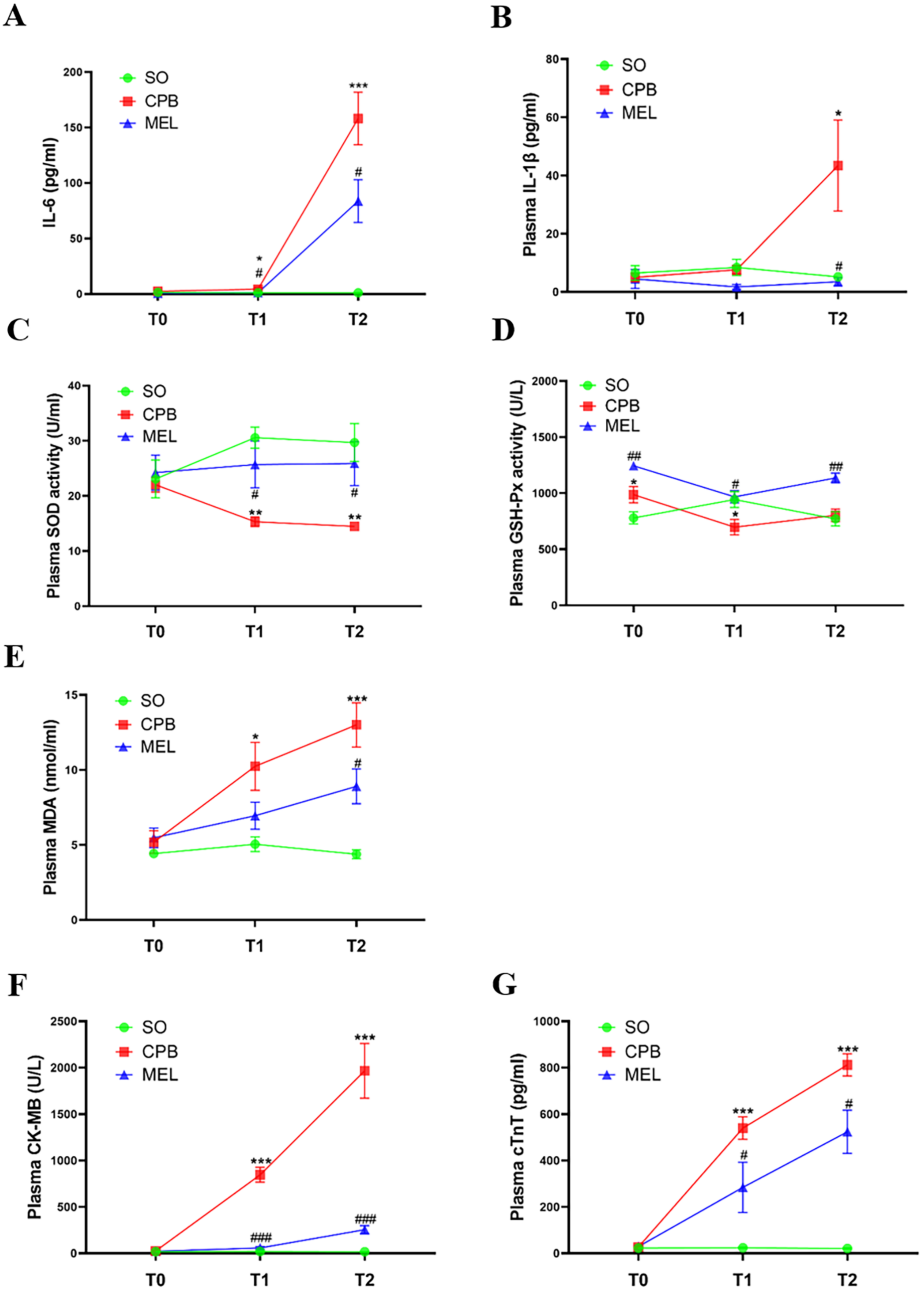
Plasma levels of pro-inflammatory cytokines, oxidative stress markers and myocardial injury markers. Plasma levels of pro-inflammatory cytokines, oxidative stress markers and myocardial injury markers. (A) IL-6 and (B) IL-1*β*; (C) SOD, (D) GSH-Px, (E) MDA; (F) CK-MB and (G) cTnT. CPB vs. SO: * *p* < 0.05, ** *p* < 0.01, *** *p* < 0.001; MEL vs. CPB: # *p* < 0.05, ## *p* < 0.01, ### *p* < 0.001. SO, sham operation; CPB, cardiopulmonary bypass; MEL, melatonin; IL: interleukin; SOD, superoxide dismutase; GSH-Px, glutathione peroxidase; MDA, malondialdehyde; CK-MB, creatine kinase-MB; cTnT, cardiac troponin T; T0: after cannulation; T1: at the end of CPB; T2: 120 min after operation. *n* = 6 for each group. Data are presented as the mean ± SEM.

All rats survived the surgical procedures. The blood biochemical indexes of the rats during the procedure are shown in Fig. 2. In the present study, we focused on the levels of pro-inflammatory cytokines (IL-6 and IL-1β), oxidative stress markers (SOD, GSH-Px and MDA) and myocardial injury markers (CK-MB and cTnT). In the CPB group, the plasma levels of IL-6 (*p* < 0.05 at T1 and *p* < 0.001 at T2, Fig. 2A), MDA (*p* < 0.05 at T1 and *p* < 0.001 at T2, Fig. 2E), CK-MB (*p* < 0.001 at both T1 and T2, Fig. 2F) and cTnT (*p* < 0.001 at both T1 and T2, Fig. 2G) were significantly increased during the procedure compared with those in the sham operation group at T1 and T2, while upregulation of IL-1β at T2 (*p* < 0.05, Fig. 2B) was observed. The plasma levels of SOD (*p* < 0.01 at both T1 and T2, Fig. 2C) in the CPB group were significantly decreased during the procedure compared with those in the sham operation group at T1 and T2, while downregulation of GSH-Px at T1 (*p* < 0.05) was observed (Fig. 2D). No abnormal heart function was observed (Supplemental Fig. 1 and Table S1). Our results were consistent with those of other previous studies (Pan et al., 2020; Song et al., 2018; Wang et al., 2018), indicating that the CPB model was successfully established in this study and could be used for further investigation. Compared with CPB alone, pretreatment with melatonin significantly reduced the levels of IL-6 (*p* < 0.05 at both T1 and T2, Fig. 2A), CK-MB (*p* < 0.001 at both T1 and T2, Fig. 2F), and cTnT (*p* < 0.05 at both T1 and T2, Fig. 2G) and increased the levels of SOD (*p* < 0.05 at both T1 and T2, Fig. 2C) and GSH-Px (*p* < 0.05 at T1 and *p* < 0.01 at T2, Fig. 2D). Moreover, melatonin decreased the levels of MDA (*p* < 0.05, Fig. 2E) and IL-1β at T2 (*p* < 0.05, Fig. 2B). These results suggested that the cardioprotective effect of melatonin may exist during CPB.

### Melatonin inhibits cardiomyocyte superoxide production and apoptosis induced by CPB

**Figure 3:**
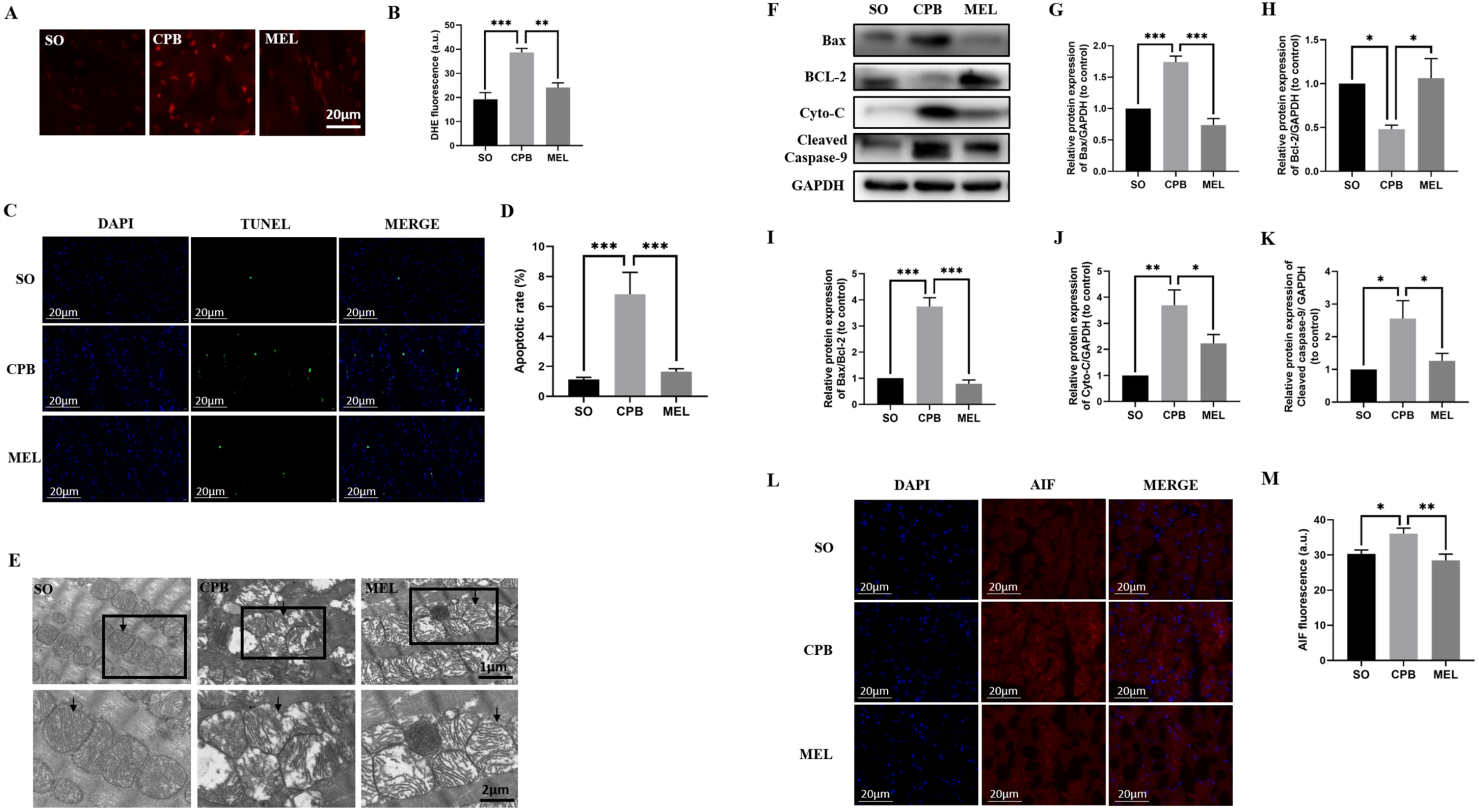
Effect of melatonin on cardiomyocyte superoxide production and apoptosis induced by CPB. Effect of melatonin on cardiomyocyte superoxide production and apoptosis induced by CPB. (A, B) The production level of ROS in fresh myocardium was detected by using DHE fluorescence (red color, 400×, *n* = 6 per group). (C, D) A TUNEL assay was used to assess cardiomyocyte apoptosis in ventricular tissue (400×, *n* = 6 per group). (E) Electron microscopic analysis of cardiomyocyte cells. CPB led to the destruction of the myocardial ultrastructure and mitochondrial swelling, which was alleviated after melatonin treatment (*n* = 3 for each group). Black arrow: mitochondria. Original magnification: 8000× and 15,000×. (F–K) Western blotting was used to assess the protein levels of Bax, BCL-2, Cyto-C, and cleaved caspase-9. GAPDH served as the internal reference (*n* = 6 for each group). (L, M) Immunofluorescence was used to detect AIF expression (*n* = 6 for each group). SO, sham operation; CPB, cardiopulmonary bypass; MEL, melatonin; ROS, reactive oxygen species; DHE, dihydroethidium; TUNEL, terminal deoxynucleotidyl transferase dUTP nick-end labeling; Bax, B cell lymphoma/leukemia2 associated X; BCL2, B cell lymphoma/leukemia2; Cyto-C, cytochrome C; GAPDH, glyceraldehyde-3-phosphate dehydrogenase; AIF, apoptosis inducing factor. Data are shown as the mean ± SEM. ^∗^*p* < 0.05, ^∗∗^*p* < 0.01, ^∗∗∗^*p* < 0.001.

Due to the alteration of MDA, SOD and GSH-Px levels in plasma, the intracellular generation of ROS in cardiomyocytes was evaluated by DHE staining. As shown in Figs. 3A and 3B, minimal fluorescence was detected in the SO group, and the fluorescence intensity was dramatically enhanced in the CPB group but was significantly decreased in the melatonin pretreatment group (SO vs. CPB, *p* < 0.001; CPB vs. MEL, *p* < 0.01).

TUNEL staining was used to detect the effect of melatonin on apoptosis induced by CPB. Our data showed that few apoptotic cardiomyocytes were detected in myocardial tissues from the SO group, but a significant number of TUNEL-positive cardiomyocytes were observed in the CPB group (*p* < 0.001. Figures 3C, 3D). Pretreatment with melatonin led to a significant antiapoptotic effect, as shown by reduced TUNEL-positive staining (*p* < 0.001. Figures 3C, 3D). Then, electron microscopic analysis of cardiomyocytes showed that mitochondrial swelling was more severe in the CPB group and was relieved after melatonin pretreatment (Fig. 3E), suggesting that the mitochondrial apoptosis pathway was involved. Furthermore, the detection of markers of mitochondrial apoptosis by western blotting confirmed this suggestion. Melatonin significantly decreased the expression of the pro-apoptotic proteins Cyto-C, Bax, cleaved caspase-9 and AIF, the expression of which were induced by CPB, and increased the expression of the anti-apoptotic protein Bcl-2, while the Bax/Bcl-2 ratio was significantly decreased (all *p* < 0.05, Figs. 3F–3M). These results suggest that melatonin might serve as a potential antiapoptotic medication by regulating Bcl-2 family proteins and cleaved caspase-9 for cardioprotection during the CPB procedure.

### Melatonin activates the AKT and STAT3 signaling pathways in cardiomyocytes during CPB

**Figure 4:**
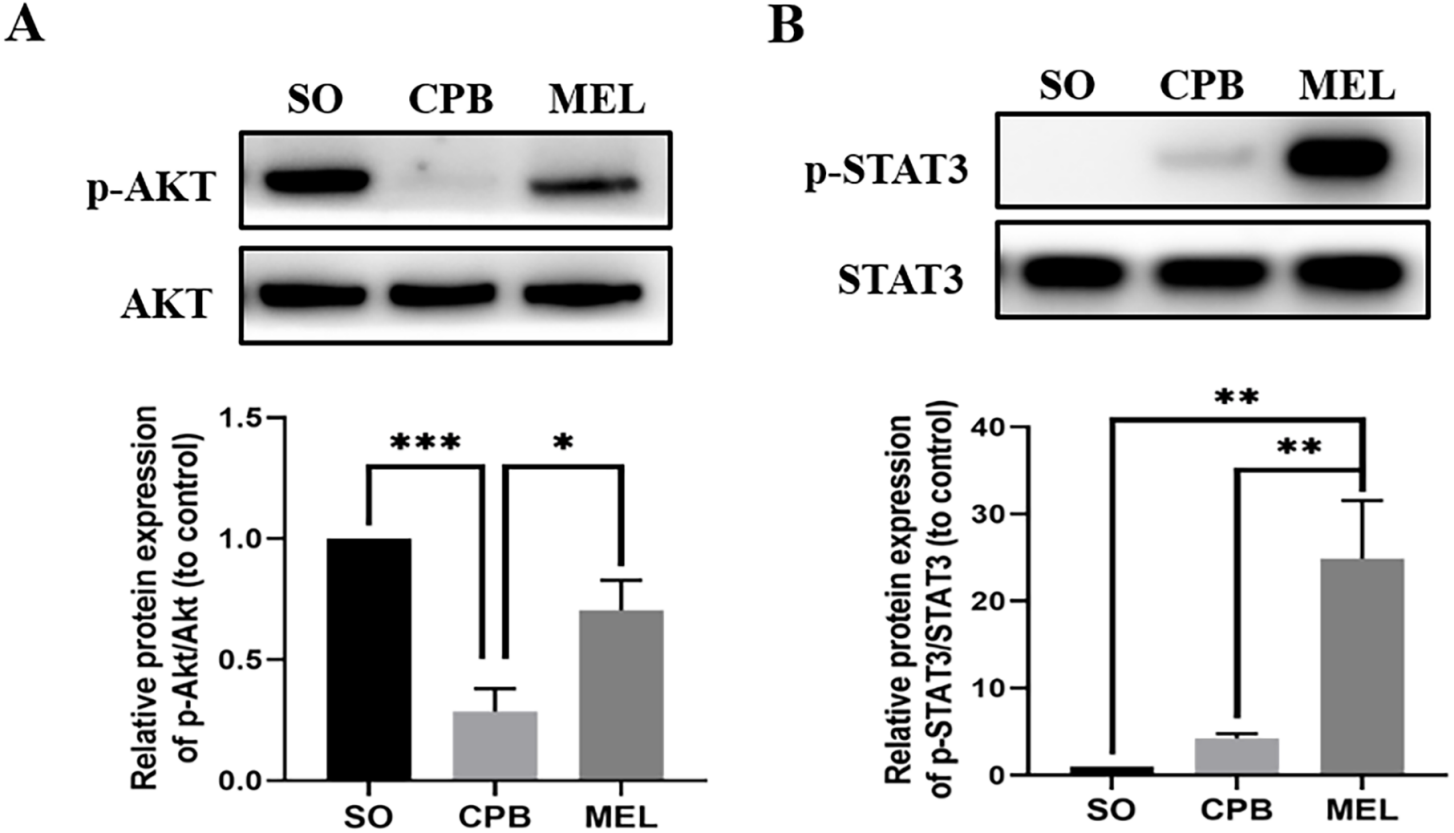
Effect of melatonin on the apoptosis signaling pathway induced by CPB. Effect of melatonin on the apoptosis signaling pathway induced by CPB. The expression and phosphorylation of (A) AKT and (B) STAT3 were detected by western blotting. SO, sham operation; CPB, cardiopulmonary bypass; MEL, melatonin; STAT3, signal transducer and activator of transcription 3; *n* = 6 for each group. Data are shown as the mean ± SEM. ^∗^*p* < 0.05, ^∗∗^*p* < 0.01, ^∗∗∗^*p* < 0.001.

Melatonin can inhibit apoptosis by regulating the AKT and STAT3 signaling pathways via specific receptors (Singhanat et al., 2018). To investigate whether these signaling pathways were involved in the anti-apoptotic effect of melatonin, we used western blotting to detect the levels of p-AKT, AKT, p-STAT3 and STAT3. As shown in Fig. 4, CPB significantly decreased the phosphorylation of AKT and slightly elevated the phosphorylation of STAT3, as shown in a previous investigation (Pan et al., 2020). Moreover, melatonin significantly increased the phosphorylation of AKT and STAT3 compared with CPB alone (all *p* < 0.05, Fig. 4). These data suggested that melatonin might inhibit apoptosis by upregulating the AKT and STAT3 signaling pathways.

### Melatonin treatment ameliorates CPB-induced autophagy of cardiomyocytes

**Figure 5:**
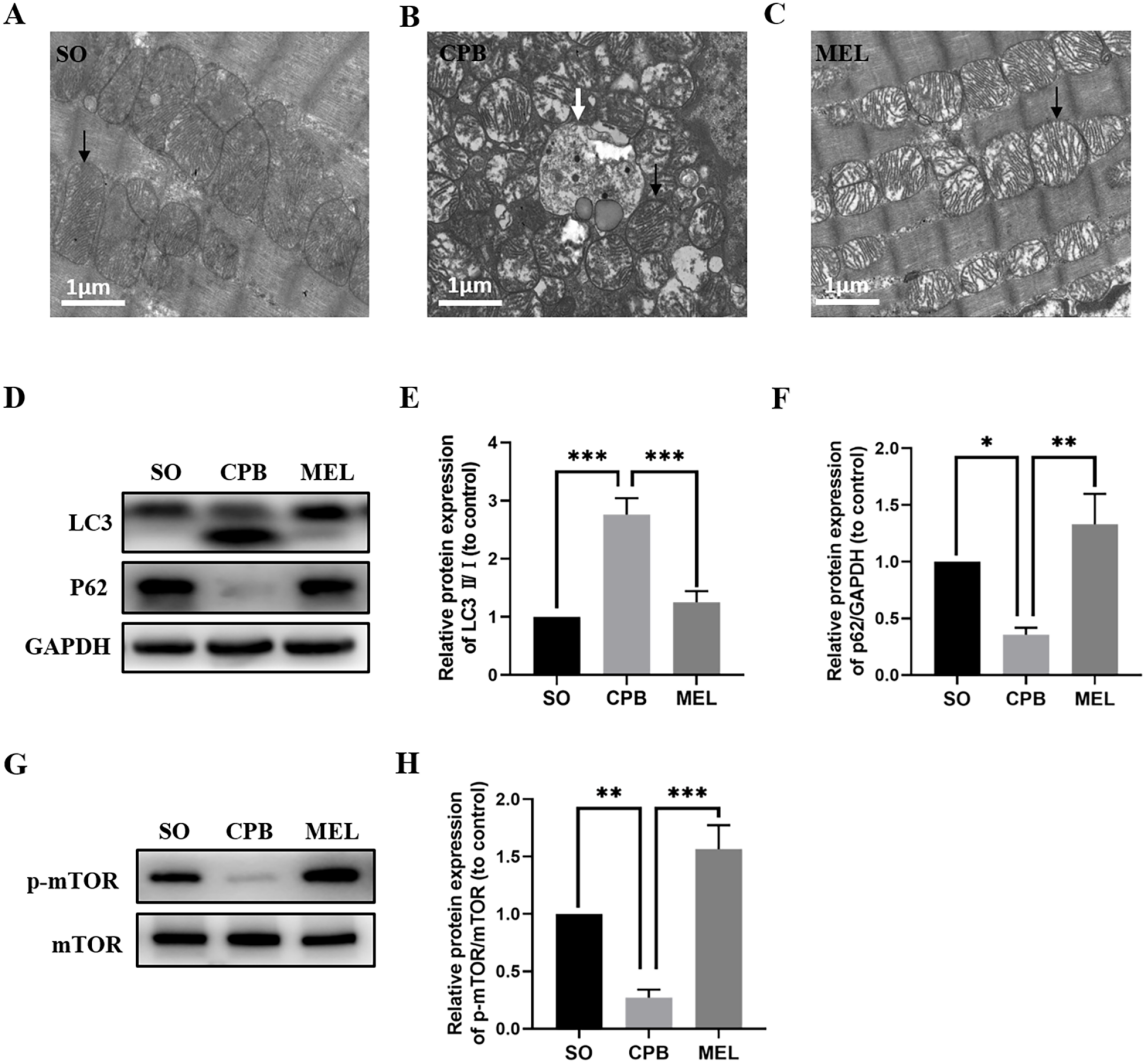
Effect of melatonin on cardiomyocyte autophagy induced by CPB. (A–C) Electron microscopic analysis of cardiomyocyte cells (*n* = 3 for each group). Western blotting was used to assess the protein levels of LC3 and P62 (D–F) as well as p-mTOR and mTOR (G, H). GAPDH served as the internal reference. Black arrow: mitochondria, white arrow: autophagosome. SO, sham operation; CPB, cardiopulmonary bypass; MEL, melatonin; mTOR, mechanistic target of rapamycin kinase; GAPDH, glyceraldehyde-3-phosphate dehydrogenase. *n* = 6 for each group. Data are shown as the mean ± SEM. ^∗^*p* < 0.05, ^∗∗^*p* < 0.01, ^∗∗∗^*p* < 0.001.

Melatonin can inhibit autophagy by interacting with its nuclear receptor ROR *α* (He et al., 2016) or by regulating the AKT/mTOR pathway (Xu et al., 2020). In the present study, we investigated whether melatonin plays a role in cardiomyocyte autophagy induced by CPB. As shown in Figs. 5A–5C, electron microscopic analysis revealed that the CPB group showed an increase in the number of autophagosomes in the heart compared with the SO group, and the number of autophagosomes in the heart was significantly decreased in the melatonin-treated group. Moreover, western blotting showed that melatonin could significantly reduce the levels of the autophagy marker LC3 while increasing the level of P62, which was altered by CPB (Figs. 5D–5F). Furthermore, melatonin could significantly increase the phosphorylation of mTOR (Figs. 5G, 5H), suggesting that the mTOR pathway was involved in the anti-autophagy effects of melatonin. Thus, these data suggested that melatonin could inhibit the autophagy of cardiomyocytes induced by CPB via the mTOR signaling pathway.

## Discussion

In the present study, we first provided in vivo evidence that continuous intraperitoneal injection of melatonin for 7 days protected the heart from injury induced by CPB. Different from the previous study of adding melatonin to the prime of CPB (Huang et al., 2008; Wang et al., 2009), we used intraperitoneal injection of melatonin. Melatonin significantly decreased the levels of myocardial injury markers (CK-MB and cTnT), oxidative stress markers (MDA) and pro-inflammatory cytokines (IL-6 and IL-1β), which were upregulated by CPB, and increased the levels of the antioxidative production of GSH-Px and SOD, which were downregulated by CPB. Moreover, melatonin inhibited cardiomyocyte superoxide production and apoptosis induced by CPB, in which the AKT and STAT3 signaling pathways might be involved. Furthermore, melatonin could inhibit the autophagy of cardiomyocytes induced by CPB, in which the mTOR signaling pathway was involved. Taken together, the results show that melatonin could serve as a cardioprotective factor in CPB by inhibiting oxidative damage, apoptosis and autophagy.

Melatonin has been recognized as a potential cardioprotective agent that may have beneficial effects on cardiovascular diseases, including hypertension, cardiac hypertrophy, heart failure and ischemic heart disease (Reiter, Tan & Galano, 2014; Yang et al., 2014). Patients with coronary heart disease have low plasma melatonin levels, especially patients with a higher risk of cardiac infarction and/or sudden death (Dominguez-Rodriguez & Abreu-Gonzalez, 2010; Dominguez-Rodriguez, Abreu-Gonzalez & Avanzas, 2012). A clinical report showed that melatonin supplementation can ameliorate myocardial ischemic-reperfusion injury by increasing the LVEF and reducing the levels of troponin-I, IL-1β, iNOS and caspase-3 (Dwaich et al., 2016). The post hoc analysis of the MARIA trial showed that the effect of melatonin was affected by the timing of reperfusion. It’s found that melatonin given <2.5 h after symptom onset could reduce myocardial infarct size by approximately 40% as measured by cardiovascular magnetic resonance. The results thus suggest that melatonin administered earlier may result in a greater cardioprotective effect compared with delayed administration and this has potential clinical implications for the treatment of patients with ST-segment elevation myocardial infarction (Dominguez-Rodriguez et al., 2017). Several studies have shown that melatonin protects against myocardial infarction (Ciosek & Drobnik, 2012), arrhythmias (Lee et al., 2002), and cardiac toxicity (Tengattini et al., 2008). Thus, melatonin can serve as a novel potential cardioprotective agent. However, few studies on its protective effects against CPB-induced myocardial injury have been reported. Our study showed that melatonin pretreatment could significantly decrease the levels of CK-MB, cTnT, MDA, IL-1β and IL-6 while increasing the levels of SOD and GSH-Px, which were altered by CPB, suggesting that melatonin pretreatment could be an effective therapy for preventing CPB-induced myocardial injury.

Melatonin can increase cell survival by activating a series of important signaling pathways, resulting in a reduction in mitochondrial and cellular oxidative stress, mitochondrial fission, endoplasmic reticulum stress, and apoptosis. Melatonin can activate p-Akt and p-STAT3 by interacting with the receptors of the SAFE, RISK and Notch1/Hes1 pathways, resulting in reduced mitochondrial oxidative stress, increased antioxidant levels, and reduced apoptosis (Nduhirabandi et al., 2016; Yu et al., 2016; Yu et al., 2015). STAT3 activation can lead to the formation of a STAT dimer that translocates into the nucleus and promotes antioxidant gene expression, leading to oxidative stress. On the other hand, STAT3 can inhibit Bax translocation, enhance the expression of the anti-apoptotic protein Bcl2, and suppress opening of the mitochondrial permeability transition pore (mPTP), resulting in cell apoptosis (Nduhirabandi et al., 2016; Yu et al., 2016). AKT activation can decrease the level of the pro-apoptotic protein Bax and enhance the production of the anti-apoptotic protein Bcl-2 (Yu et al., 2015). Next, the activation of caspase promotes the release of cytochrome C and AIF, which mediate the mitochondrial regulation of apoptosis (Ola, Nawaz & Ahsan, 2011). Our study showed that melatonin can reduce intracellular ROS generation, the TUNEL-stained cell percentage, and the mitochondrial swelling of cardiomyocytes during CPB (Figs. 3A– 3E), which might result from the alteration of Bax, Bcl-2, Cyto-C, AIF and Caspase-9, which are regulated by the AKT and STAT3 pathways (Figs. 3F–3M, 4A, 4B). Taken together, the results suggest that melatonin can serve as an antiapoptotic medication for cardioprotection during CPB.

Autophagy, an important degradation process that participates in the turnover of damaged intracellular cytosolic proteins and organelles that is dependent on lysosomes, is critical for the maintenance of normal cell function (Füllgrabe et al., 2014). CPB can cause myocardial injury by altering myocardial autophagy (Hua et al., 2017). In the present study, we observed that CPB dramatically increased the accumulation of autophagosomes and enhanced LC3 levels while decreasing the p62 level, suggesting the presence of excessive autophagy (Figs. 5A–5F). Our study also showed that melatonin can significantly decrease the number of autophagosomes and the level of LC3 and enhance p62 levels (Figs. 5A–5F), which is consistent with previous findings (Xu et al., 2020). Melatonin can inhibit autophagy by interacting with its nuclear receptor ROR *α* (He et al., 2016) or by regulating the AKT/mTOR pathway (Xu et al., 2020). In our study, we found that p-mTOR was downregulated in the CPB group but was significantly upregulated in the melatonin treatment group (Figs. 5G, 5H), suggesting that mTOR signaling was involved in the resistance to autophagy induced by melatonin during CPB. Thus, melatonin can ameliorate myocardial injury by attenuating autophagy via the mTOR pathway during CPB.

There are several potential limitations of this study that need to be discussed. First, we only investigated the protective effect of melatonin on myocardial injury induced by cardiopulmonary bypass, and did not study the adverse effects of melatonin. Second, we used CPB in this study but did not perform aortic cross clamp and cardioplegic arrest in this study due to the technical limits. Finally, we injected melatonin intraperitoneally rather than orally. In terms of pharmacokinetics, previous study has shown that the bioavailability of oral melatonin at 10 mg/kg in rats is 53.5%, while the bioavailability of melatonin at the same concentration by intraperitoneal injection is 74%. Because of the first pass effect of the liver, the bioavailability of oral melatonin in rats is relatively lower, while intraperitoneal and intravenous injections of melatonin in rats have higher bioavailability (Yeleswaram et al., 1997). For better clinical application, we will compare the therapeutic effects of oral, intravenous and intraperitoneally.

## Conclusions

In summary, melatonin significantly decreased the levels of myocardial injury markers (CK-MB and cTnT), oxidative stress markers (MDA) and pro-inflammatory cytokines (IL-6 and IL-1β). On the contrary, melatonin increased the levels of the oxidative stress markers SOD and GSH-Px during CPB. Melatonin inhibited cardiomyocyte superoxide production and apoptosis via the AKT and STAT3 pathways in CPB. Melatonin could inhibit the autophagy of cardiomyocytes via the mTOR pathway in CPB. Taken together, the results indicate that melatonin may serve as a cardioprotective factor in CPB by inhibiting oxidative damage, apoptosis and autophagy.

##  Supplemental Information

10.7717/peerj.11264/supp-1 Supplemental Information 1 Echocardiographic parameters and effect of melatonin on echocardiography alteration induced by CPB

10.7717/peerj.11264/supp-2 Supplemental Information 2 The ARRIVE guidelines 2.0: author checklist

10.7717/peerj.11264/supp-3 Supplemental Information 3 Raw data for Fig. 3

10.7717/peerj.11264/supp-4 Supplemental Information 4 Raw data for Fig. 4

10.7717/peerj.11264/supp-5 Supplemental Information 5 Raw data for Fig. 5

10.7717/peerj.11264/supp-6 Supplemental Information 6 Raw data for all figures

## Additional Information and Declarations

### Data Availability

The following information was supplied regarding data availability: The raw data is available in the Supplementary File.
