# Supplementary material for: Melatonin ameliorates myocardial injury by reducing apoptosis and autophagy of cardiomyocytes in a rat cardiopulmonary bypass model
Source: PeerJ. 2021 Apr 15;9:e11264. doi: 10.7717/peerj.11264 (PMC8053380; doi:10.7717/peerj.11264)
Supplement: Supplemental Information 1 [file peerj-09-11264-s001.doc]

**SUPPLEMENTAL MATERIALS**

**Supplement Table 1. Echocardiographic parameters.**

| **Variables** | **SO** | **CPB** | **MEL** |
| --- | --- | --- | --- |
| **LVEF (%)** |  |  |  |
| T0 | 75.59 ± 3.60 | 74.98 ± 3.85 | 76.92 ± 4.20 |
| T1 | 75.97 ± 0.47 | 77.88 ± 3.83 | 75.16 ± 3.42 |
| T2 | 77.12 ± 0.76 | 69.94 ± 3.75 | 76.36 ± 4.18 |
| **LVFS (%)** |  |  |  |
| T0 | 45.94 ± 3.46 | 45.48 ± 3.77 | 47.37 ± 3.87 |
| T1 | 46.00 ± 2.97 | 47.96 ± 3.51 | 45.23 ± 3.05 |
| T2 | 48.18 ± 1.55 | 40.76 ± 3.03 | 47.08 ± 4.14 |
| **IVS;d (mm)** |  |  |  |
| T0 | 2.27 ± 0.09 | 2.13 ± 0.10 | 2.30 ± 0.09 |
| T1 | 2.25 ± 0.11 | 2.21 ± 0.10 | 2.20 ± 0.16 |
| T2 | 2.11 ± 0.04 | 2.60 ± 0.06 | 2.21 ± 0.16 |
| **IVS;s (mm)** |  |  |  |
| T0 | 3.39 ± 0.13 | 3.34 ± 0.17 | 3.50 ± 0.15 |
| T1 | 3.51 ± 0.09 | 3.47 ± 0.13 | 3.39 ± 0.18 |
| T2 | 3.33 ± 0.07 | 3.50 ± 0.26 | 3.63 ± 0.12 |
| **LVID;d (mm)** |  |  |  |
| T0 | 6.21 ± 0.34 | 6.23 ± 0.23 | 6.13 ± 0.21 |
| T1 | 6.51 ± 0.18 | 5.79 ± 0.28 | 5.94 ± 0.22 |
| T2 | 6.40 ± 0.20 | 6.24 ± 0.12 | 6.42 ± 0.24 |
| **LVID;s (mm)** |  |  |  |
| T0 | 3.37 ± 0.34 | 3.40 ± 0.28 | 3.25 ± 0.31 |
| T1 | 3.54 ± 0.27 | 3.04 ± 0.30 | 3.27 ± 0.27 |
| T2 | 3.32 ± 0.13 | 3.71 ± 0.24 | 3.38 ± 0.24 |
| **LVPW;d (mm)** |  |  |  |
| T0 | 2.80 ± 0.38 | 3.12 ± 0.51 | 2.38 ± 0.42 |
| T1 | 2.44 ± 0.23 | 2.21 ± 0.12 | 2.80 ± 0.42 |
| T2 | 2.76 ± 0.22 | 2.42 ± 0.23 | 2.45 ± 0.13 |
| **LVPW;s (mm)** |  |  |  |
| T0 | 4.07 ± 0.29 | 4.08 ± 0.35 | 3.51 ± 0.38 |
| T1 | 3.30 ± 0.30 | 3.12 ± 0.22 | 3.92 ± 0.34 |
| T2 | 3.63 ± 0.26 | 3.26 ± 0.26 | 3.97 ± 0.17 |
| **LV Mass (mg)** |  |  |  |
| T0 | 1282.00 ± 181.19 | 1346.48 ± 140.19 | 1101.97 ± 119.01 |
| T1 | 1176.75 ± 64.25 | 918.55 ± 47.96 | 3199.63 ± 2047.49 |
| T2 | 1220.35 ± 56.43 | 1258.37 ± 92.50 | 1176.78 ± 81.51 |
| **LV Vol;d (μL)** |  |  |  |
| T0 | 183.87 ± 27.45 | 197.61 ± 16.86 | 190.95 ± 15.33 |
| T1 | 213.98 ± 9.26 | 168.87 ± 17.31 | 177.70 ± 15.20 |
| T2 | 222.88 ± 4.06 | 197.52 ± 8.32 | 216.03 ± 17.65 |
| **LV Vol;s (μL)** |  |  |  |
| T0 | 51.69 ± 10.63 | 50.03 ± 9.69 | 45.77 ± 10.24 |
| T1 | 51.36 ± 2.26 | 39.06 ± 8.64 | 45.69 ± 8.84 |
| T2 | 51.03 ± 2.08 | 60.52 ± 9.27 | 49.94 ± 8.72 |

Data were expressed as the mean ± SEM. T0: after cannulation; T1: at the end of CPB; T2: 120 min after operation. SO: sham operation; CPB: cardiopulmonary bypass; MEL: melatonin; LVEF: ejection fraction of the left ventricle; LVFS: fractional shortening of the left ventricle; IVSd: end-diastolic interventricular septum; IVSs: end-systolic interventricular septum; LVIDd: end-diastolic left ventricular internal diameter; LVIDs: end-systolic left ventricular internal diameter; LVPWd: end-diastolic left ventricular posterior wall; LVPWs: end-systolic left ventricular left ventricular posterior wall; LV Mass: left ventricular mass; LV Vold: end-diastolic left ventricular volume; LV Vols: end-systolic left ventricular volume. n=6 per group.


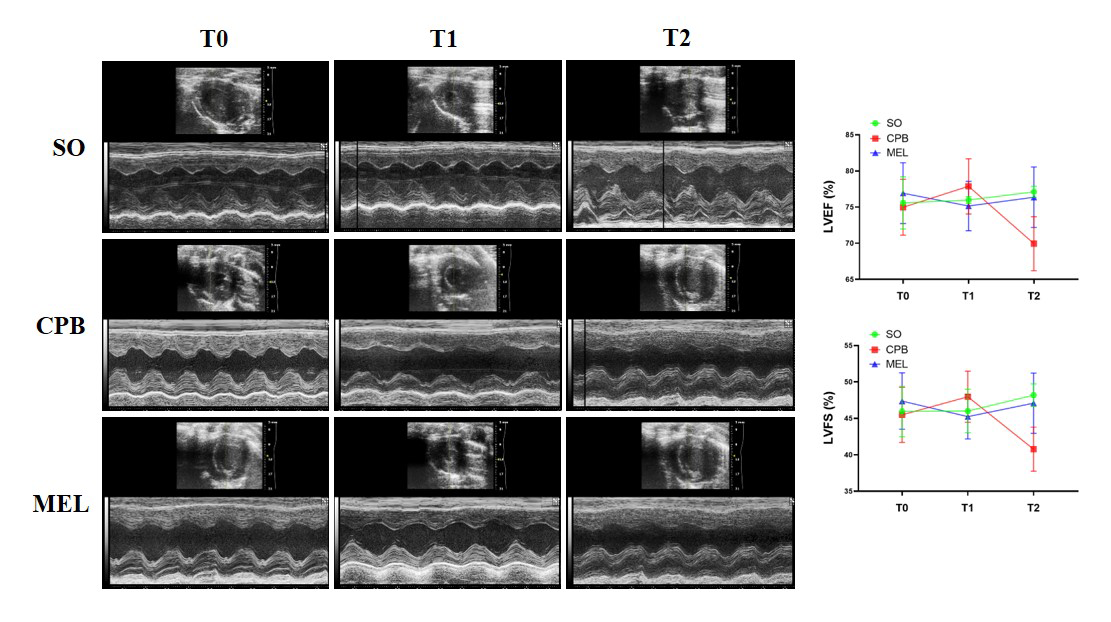


**Supplement figure S1.** Effect of melatonin on echocardiography alteration induced by CPB. Echocardiography performed after cannulation (T0), the end of CPB (T1), 120 min after operation (T2). Left ventricular ejection fraction (LVEF) and fractional shortening (LVFS) in the CPB group were lower than those in the other two groups at T2 but these differences were not statistically significant (p ≥ 0.05). SO: sham operation; CPB: cardiopulmonary bypass; MEL: melatonin. n = 6 for each group. Data are shown as the mean ± SEM.
